# Nausea from Chloroform Anesthesia

**Published:** 1898-02

**Authors:** 


### Nausea from Chloroform Anesthesia.

When an operation under chloroform has been finished, pour vinegar upon the mask until it is well saturated, and leave the mask in place. As the vinegar evaporates more should be added. This simple procedure has a marked effect in preventing or modifying the nausea after chloroform anesthesia. It was first advised by a French surgeon who says that it acts by the vinegar's forming a non-irritating combination with the chloroform vapor already changed in the lungs.—*International Journal of Surgery.*

